# Phenotypic and Molecular Characteristics of Carbapenem-Resistant *Acinetobacter baumannii* Isolates from Bulgarian Intensive Care Unit Patients

**DOI:** 10.3390/microorganisms11040875

**Published:** 2023-03-29

**Authors:** Tanya V. Strateva, Ivo Sirakov, Temenuga J. Stoeva, Alexander Stratev, Slavil Peykov

**Affiliations:** 1 Department of Medical Microbiology, Faculty of Medicine, Medical University of Sofia, 2 Zdrave Str., 1431 Sofia, Bulgaria; 2 Department of Microbiology and Virology, Faculty of Medicine, Medical University of Varna, 55 Marin Drinov Str., 9002 Varna, Bulgaria; 3 Intensive Care Unit, University Multiprofile Hospital for Active Treatment ‘St. Ivan Rilski’, 15 Acad. Ivan Geshov Blvd., 1431 Sofia, Bulgaria; 4 Department of Anaesthesiology and Intensive Care, Faculty of Medicine, Medical University of Sofia, 1 St. Georgi Sofiyski Str., 1431 Sofia, Bulgaria; 5 Department of Genetics, Faculty of Biology, University of Sofia ‘St. Kliment Ohridski’, 8 Dragan Tzankov Blvd., 1164 Sofia, Bulgaria; 6 BioInfoTech Laboratory, Sofia Tech Park, 111 Tsarigradsko Shose Blvd., 1784 Sofia, Bulgaria

**Keywords:** *Acinetobacter baumannii*, carbapenem resistance, extensive drug resistance, antimicrobial resistance genes, whole-genome sequencing, resistome analysis

## Abstract

Carbapenem-resistant *Acinetobacter baumannii* (CRAB) is designated as an urgent public health threat, both due to its remarkable multidrug resistance and propensity for clonal spread. This study aimed to explore the phenotypic and molecular characteristics of antimicrobial resistance in CRAB isolates (*n* = 73) from intensive care unit (ICU) patients in two university hospitals in Bulgaria (2018–2019). The methodology included antimicrobial susceptibility testing, PCR, whole-genome sequencing (WGS), and phylogenomic analysis. The resistance rates were as follows: imipenem, 100%; meropenem, 100%; amikacin, 98.6%; gentamicin, 89%; tobramycin, 86.3%; levofloxacin, 100%; trimethoprim–sulfamethoxazole, 75.3%; tigecycline, 86.3%; colistin, 0%; and ampicillin–sulbactam, 13.7%. All isolates harbored *bla*_OXA-51-like_ genes. The frequencies of distribution of other antimicrobial resistance genes (ARGs) were: *bla*_OXA-23-like_, 98.6%; *bla*_OXA-24/40-like_, 2.7%; *armA*, 86.3%; and *sul1*, 75.3%. The WGS of selected extensively drug-resistant *A. baumannii* (XDR-AB) isolates (*n* = 3) revealed the presence of OXA-23 and OXA-66 carbapenem-hydrolyzing class D β-lactamases in all isolates, and OXA-72 carbapenemase in one of them. Various insertion sequencies, such as IS*Aba24*, IS*Aba31*, IS*Aba125*, IS*Vsa3*, IS*17*, and IS*6100*, were also detected, providing increased ability for horizontal transfer of ARGs. The isolates belonged to the widespread high-risk sequence types ST2 (*n* = 2) and ST636 (*n* = 1) (Pasteur scheme). Our results show the presence of XDR-AB isolates, carrying a variety of ARGs, in Bulgarian ICU settings, which highlights the crucial need for nationwide surveillance, especially in the conditions of extensive antibiotic usage during COVID-19.

## 1. Introduction

*Acinetobacter baumannii* belongs to a group of antimicrobial-resistant nosocomial pathogens, designated by the acronym “ESKAPE” (*Enterococcus faecium*, *Staphylococcus aureus*, *Klebsiella pneumoniae*, *Acinetobacter baumannii*, *Pseudomonas aeruginosa*, and *Enterobacter* spp.). These bacteria represent a substantial therapeutic problem worldwide [1]. They frequently “escape” from the most commonly used antimicrobial treatment via acquisition and/or development of various resistance mechanisms [2].

Over the last decades, *A. baumannii* has globally emerged as a highly troublesome nosocomial pathogen [3], particularly in intensive care unit (ICU) settings, where it causes a wide array of healthcare-associated infections (HAIs) and long-term outbreaks, such as ventilator-associated pneumonia (VAP), bloodstream infection (BSI), urinary tract infection, wound infection and meningitis [4,5,6,7,8]. However, community-acquired *A. baumannii* infections have been described, in particular, in individuals with comorbidities [9,10,11]. Additionally, recent studies have reported that respiratory viral infections, such as severe acute respiratory syndrome coronavirus 2 (SARS-CoV-2), predispose patients to bacterial co-infections and secondary infections [12]. Prolonged ICU stay may result in increased possibility of developing bacterial co-infection, especially with multidrug-resistant *A. baumannii* (MDR-AB) in critically ill coronavirus disease 2019 (COVID-19) patients [13,14].

*A. baumannii* infections are usually hard to treat owing to innate and acquired resistance to multiple antimicrobial agents. Carbapenems, recognized as the therapy of choice for serious HAIs and for the care of patients affected by MDR-AB, are no longer effective in some cases [15,16]. Carbapenem-resistant *A. baumannii* (CRAB) is designated as an urgent public health threat by the Centers for Disease Control and Prevention [17]. Its increasing importance has been recognized by the World Health Organization (WHO), which classified CRAB amongst the critical-priority pathogens in the ‘Global priority list of antibiotic-resistant bacteria to guide research, discovery, and development of new antibiotics, 2017’ [18]. Recent trends reveal that many infections are caused by CRAB, MDR-AB, extensively drug-resistant (XDR-AB) or even pan drug-resistant *A. baumannii* (PDR-AB), for which effective treatment options are severely limited [16,19,20].

Carbapenem resistance in *A. baumannii* is predominantly related to the production of carbapenem-hydrolyzing β-lactamases (carbapenemases), and rarely to the loss or modification of outer membrane proteins and/or the overexpression of active efflux pump systems [16,21]. In addition to the naturally occurring class D β-lactamases, also called oxacillinases (OXAs), several acquired carbapenem-hydrolyzing class D β-lactamases (CHDLs) have been identified in *A. baumannii*, among which OXA-23-like enzymes are the most common [22]. Other commonly found CHDLs include OXA-24/40-like and OXA-58-like enzymes [23]. It is worth mentioning that most acquired CHDLs possess weak carbapenemase activity, but the presence of specific insertion sequences (ISs) upstream of the CHDL gene leads to their overexpression, conferring carbapenem resistance [24]. A variety of class B metallo-β-lactamases (MBLs) have been identified in *A. baumannii*, but these occur at a much lower frequency than CHDLs. Class A serine β-lactamases with carbapenemase activity have been reported occasionally [3,25]. Multiple reports have demonstrated higher resistance percentages of CRAB in Southern and Eastern Europe than Northern Europe, as well as very high carbapenem resistance rates in the Balkan countries [26].

In addition to its remarkable multidrug resistance, *A. baumannii* is characterized by a propensity for clonal spread. During the past five decades, the evolution of *A. baumannii* was mainly driven by two global clones or international clones (ICs) of high risk (IC1 and IC2) [27]. Molecular epidemiological studies have assigned nine ICs (IC1–9) [28,29], the most widespread of which is IC2, commonly harboring the acquired OXA-23 carbapenemase [28,30,31].

The aim of this study was to explore the phenotypic and molecular characteristics of antimicrobial resistance in CRAB isolates from ICU patients treated at two large hospitals in Bulgaria (2018–2019). A phylogenomic analysis was also performed.

## 2. Materials and Methods

### 2.1. Bacterial Isolates

A collection of 73 non-duplicate carbapenem-resistant MDR-AB isolates was studied. The isolates were collected during the period of January 2018 to December 2019 from ICU patients (29 females and 44 males; age range 5–86 years) treated at two multi-profile university hospitals in Bulgaria, namely the University Hospital ‘St. Ivan Rilski’, Sofia (405 beds) (SIR-S), and the University Hospital ‘St. Marina’, Varna (1416 beds) (SM-V). The isolates were obtained from tracheobronchial aspirate (*n* = 44), throat secretions (*n* = 10), surgical wounds or abscesses (*n* = 7), blood (*n* = 4), cerebrospinal fluid (*n* = 4), urine (*n* = 2), and central venous catheters (*n* = 2).

*A. baumannii* ATCC 19,606 was used as a control strain for species identification, antimicrobial susceptibility testing and quinolone-resistance-determining regions (QRDR) analysis.

All procedures involving patients were performed in accordance with the ethical standards of the Medical University of Sofia, Bulgaria, and the Helsinki Declaration of 1964 and its later amendments. The current study was focused solely on bacterial isolates and no personal patient information was used; therefore, formal consent was not required.

### 2.2. Species Identification of the Isolates

Species identification was done using the BD Phoenix M50 automated system (BD, Franklin Lakes, NJ, USA). Confirmation of species identity was performed by a polymerase chain reaction (PCR)-based *gyrB* method, as described previously [32]. The method distinguished between the closely related species *A. baumannii* and *Acinetobacter nosocomialis*. The identification of three selected isolates was further confirmed by analyzing the assembled draft genome sequence using the Microbial Genomes Atlas (MiGA) Web server [33]. The included workflow for the National Center for Biotechnology Information (NCBI) Genome Database, prokaryotic section was followed with default settings.

### 2.3. Antimicrobial Susceptibility Testing

The antimicrobial susceptibility of the investigated *A. baumannii* isolates was determined by the minimum inhibitory concentration (MIC) gradient test (MIC Test Strip; Liofilchem, Roseto degli Abruzzi, Italy) or the broth microdilution method (Sensi Test Colistin; Liofilchem), according to the European Committee on Antimicrobial Susceptibility Testing (EUCAST) recommendations [34], to the following antimicrobial agents: imipenem (IPM), meropenem (MEM), amikacin (AMK), gentamicin (GEN), tobramycin (TOB), levofloxacin (LVX), trimethoprim–sulfamethoxazole (SXT), tigecycline (TGC), and colistin (COL). The EUCAST MIC breakpoints listed for *Enterobacterales* were applied to interpret TGC susceptibility (MICs ≤ 0.5 mg/L denoting susceptibility and >0.5 mg/L denoting resistance). In addition, susceptibility to ampicillin–sulbactam (SAM) was tested by the disk diffusion method and the results were interpreted according to the Clinical and Laboratory Standards Institute (CLSI) guidelines [35].

### 2.4. Definition of MDR-AB, XDR-AB and PDR-AB Isolates

According to previously described criteria [36], MDR-AB isolates were non-susceptible to at least one agent in three or more categories, whereas XDR-AB isolates were non-susceptible to at least one agent in all but two or fewer categories, including aminoglycosides (GEN, AMK, TOB), antipseudomonal carbapenems (IPM, MEM), antipseudomonal fluoroquinolones (ciprofloxacin, LVX), penicillins + β-lactamase inhibitors (SAM), folate pathway inhibitors (SXT), polymyxins (COL, poly- myxin B) and tetracyclines. PDR-AB isolates were non-susceptible to all antimicrobial agents listed.

### 2.5. DNA Isolation

Total DNA from all investigated strains was isolated by the DNeasy Blood and Tissue Kit (QIAGEN, Hilden, Germany), according to the manufacturer’s instructions, from 3 mL of overnight cultures inoculated with a single colony.

### 2.6. PCR-Based Screening for Antimicrobial Resistance Genes (ARGs)

PCR was performed to detect the presence of genes encoding different CHDLs (OXA enzymes), IS*Aba1*, MBLs, KPC-type class A carbapenemases, ArmA 16S rRNA methylase, and dihydropteroate synthase type I. Oligonucleotides used as primers for amplification were synthesized by Metabion (Planegg, Germany) and are listed in Table 1 [37,38,39,40,41,42,43,44]. Each 25 μL PCR mixture consisted of 2 μL of template DNA, 0.1 μM of each primer, 12.5 μL of MyTaq PCR mix (Bioline, London, UK) and 8.5 μL of ultrapure 18.2 MΩ PCR water (Bioline).

DNA was amplified in a Gene ProThermal Cycler (Bioer Technology, Hangzhou, China) using the following protocol: initial denaturation at 95 °C for 5 min, followed by 30 cycles of denaturation at 95 °C for 45 s, annealing at 50–60 °C for 45 s and extension at 72 °C for 45 s, and a single final extension at 72 °C for 7 min. PCR products were separated in 1.5% agarose gel for 50 min at 130 V, stained with SimplySafe (0.05 µL/mL) (EURx, Gdansk, Poland) and detected by ultraviolet light (wavelength 312 nm). Amplified genes were identified on the basis of fragment size (Table 1).

### 2.7. Whole-Genome Sequencing (WGS)

Three XDR-AB isolates (Aba52, Aba176 and Aba190) were subjected to WGS in order to perform a resistome analysis. The inclusion criteria were: different place of isolation (Sofia and Varna), different year of isolation and origin (regarding the isolates from Sofia), different level of aminoglycoside resistance, different ARGs previously determined by PCR. Furthermore, these were the only three isolates in which IS*Aba1* was not identified using PCR. The WGS was carried out using DNA nanoball sequencing technology, as previously described [45]. Briefly, genomic DNA obtained from all selected strains was randomly fragmented using a Covaris g-TUBE device, and fragments were size-selected by magnetic beads to an average size of 200 to 400 bp. The purified fragments from each sample were end-repaired, 3′-adenylated, ligated to adapters, and, then, PCR-amplified. All libraries generated in this way were then uploaded onto an MGISEQ-2000 platform (BGI Group, Hong Kong, China). The following sequencing step was done generating 2 × 150 bp paired-end reads.

### 2.8. Draft Genome Assembly

All steps of quality control, raw reads preprocessing and draft genome assembly were carried out through the Galaxy online platform [46,47]. Default parameters were used for all following software tools, unless otherwise specified. The entire procedure was performed using the following tools: FastQC v0.11.9, Trimmomatic v0.38 and SPAdes v3.12.0.

### 2.9. Resistome and Multilocus Sequence Typing (MLST) Analyses

The draft genome contigs were screened for ARGs using the ABRicate tool (Galaxy Version 1.0.1) with the following settings: NCBI Bacterial Antimicrobial Resistance Reference Gene Database, Minimum DNA identity (70%) and Minimum DNA coverage (60%). The read-mapping against the pA105-2 plasmid was performed applying the Bowtie2 tool (Galaxy version 2.5.0) and then visualized by the BLAST Ring Image Generator (BRIG) program [48]. All mobile genetic elements were detected using the MobileElementFinder tool with default settings [49]. Afterwards, MLST was done on the assembled draft genome sequences using the MLST tool (Galaxy Version 2.19.0, https://usegalaxy.eu/, accessed on 18 January 2023). Classification of the isolates into sequence types (ST) was done according to the Pasteur scheme. This scheme includes the identification of seven internal housekeeping genes: *cpn60* (60-kDa chaperonin), *fusA* (elongation factor EF-G), *gltA* (citrate synthase), *pyrG* (CTP synthase), *recA* (homologous recombination factor), *rplB* (50S ribosomal protein L2) and *rpoB* (RNA polymerase subunit B) [50].

### 2.10. Phylogenomic Analysis

A set of 66 genomes of colistin-susceptible *A. baumannii* isolates (Table S1) were uploaded to the KBase platform [51], together with our WGS-analyzed isolates (Aba52, Aba176 and Aba190). Then, all sequences were annotated with Prokka (v1.14.5) and SpeciesTree (v2.2.0) with default settings, used to build a phylogenetic tree of all isolates based on a collection of 49 core universal genes defined by COG (cluster of orthologous groups) gene families.

### 2.11. Statistical Analysis

The antimicrobial resistance rates of the studied *A. baumannii* isolates were compared with recent results reported by other authors, using the Student’s *t*-test. For simple comparison tests, a *p*-value of <0.05 was considered statistically significant. To counteract the problem of multiple comparisons, when used, a Bonferroni correction was applied.

## 3. Results

### 3.1. Antimicrobial Susceptibility of the Investigated A. baumannii Isolates

The antimicrobial resistance rates were as follows: IMP, 100%; MEM, 100%; AMK, 98.6%; GEN, 89%; TOB, 86.3%; LVX, 100%; SXT, 74%; TGC, 86.3%; COL, 0%; and SAM, 13.7% (Table S2). The highest activity against the studied CRAB isolates demonstrated COL > SAM > SXT > TOB and TGC. A total of 16 isolates (21.9%) were classified as MDR-AB and the remaining 57 isolates (78.1%) possessed an XDR resistance profile, with 13 of them (17.8%) showing resistance to all antibiotics tested except COL.

### 3.2. PCR Screening for ARGs

The frequencies of distribution of the most prevalent CHDLs among the investigated CRAB isolates were as follows: OXA-51-like, 100%; OXA-23-like, 97.3%; OXA-24/40-like, 1.4%; and combined OXA-23-like + OXA-24/40-like, 1.4%. The only two isolates that were identified as *bla*_OXA-24/40-like_-carrying strains (alone or in combination with *bla*_OXA-23-like_) were obtained from the hospital in Varna (SM-V). The isolates studied were negative for other CHDL genes, such as *bla*_OXA-58-like_, *bla*_OXA-143-like_ and *bla*_OXA-235-like_. None of the CRAB isolates harbored MBL genes (*bla*_VIM_, *bla*_IMP_ and *bla*_NDM_), as well as genes encoding KPC-type class A carbapenemases. IS*Aba1* insertion sequencies were found in 95.9% of the isolates.

A total of 63 of the 73 studied CRAB isolates (86.3%) revealed high-level resistance to AMK, GEN and TOB (MICs > 256 mg/L), and were later identified as ArmA and OXA-23 co-producers. SXT-resistant *A. baumannii* isolates (75.3%) were shown to be *sul1*-positive.

### 3.3. Draft Genome Assemblies: Evaluation and Comparison

Three XDR-AB isolates (Aba52, Aba176 and Aba190) were subjected to WGS-based resistome analysis. The phenotypic and genotypic characteristics of the selected isolates are summarized in Table 2.

The three assembled draft genomes varied in size between 3.96 and 4.14 Mbp, and their GC content was 38.9% (Table 3). 

### 3.4. Resistome Analysis

All identified ARGs found in the three sequenced genomes are listed in Table 4. Moreover, a wide variety of ISs were identified in all analyzed isolates, while only the Aba190 strain harbored a single Tn*6018* transposon.

The 5′ ends of the *bla*_ADC-25_ (encoding class C extended-spectrum β-lactamase ADC-25) and *bla*_OXA-23_ β-lactam resistance determinants were always found next to a contig end, suggesting the presence of ISs at the start of the corresponding promotor regions. 

**Figure 1:**
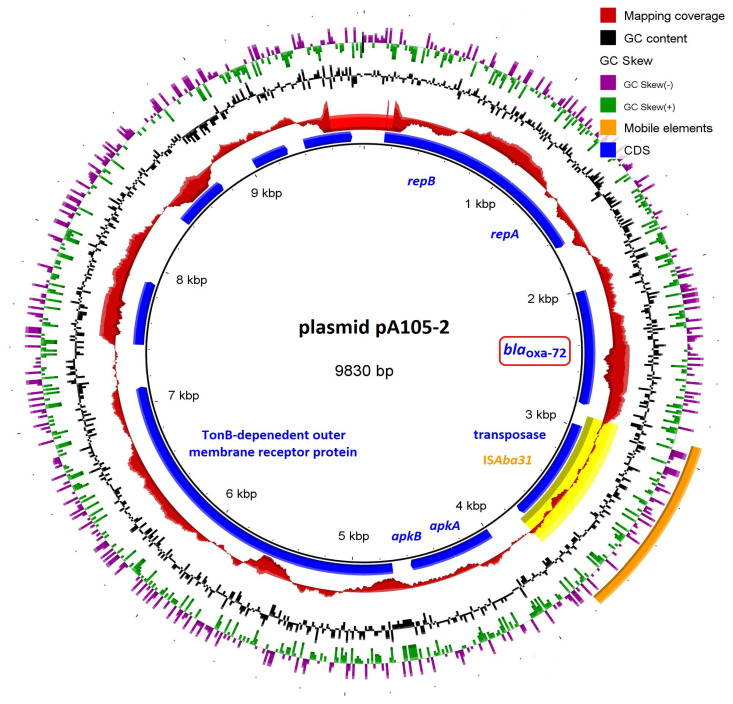
*A. baumannii* pA105−2 plasmid, showing read-mapping from the sequencing reads, CDS and mobile genetic elements. Alignments were performed using BLAST+. The image was generated using BRIG. CDS; coding sequences.

Isolate Aba190 was found to harbor a *bla*_OXA-72_ gene. The sequencing coverage of its corresponding contig was more than six times higher compared to the contig including the *bla*_OXA-66_ gene with a known chromosomal location. Such coverage bias suggested that *bla*_OXA-72_ was located on a plasmid. This hypothesis was also supported by the fact that just next to it, two genes encoding for proteins involved in the replication of DNA were found. Blast search with the 3785 bp long contig generated a perfect hit (100% query coverage and identity) against the *bla*_OXA-72_ -carrying plasmid pA105-2 (KR535993.1), isolated from the *A. baumannii* A105 strain. Mapping the sequencing reads against its sequence showed that Aba190 also possessed the remaining plasmid elements, as illustrated in Figure 1.

A variety of structural genes encoding aminoglycoside-modifying enzymes were identified in the WGS-subjected XDR-AB isolates. Moreover, *armA* genes were found in the isolates from Sofia (Aba52 and Aba176), which is in accordance with their high-level aminoglycoside resistance.

All isolates investigated were positive for *sul1*, *qacEΔ1* and *intI1* genes, suggesting the presence of class 1 integrons. The complete resistance gene cassettes embedded into the variable regions were only partially recovered due to the limitations of the used sequencing technology with a short reading frame. They contained *aac(3)-Ia* and *ant(3″)-Ia* genes, encoding for aminoglycoside-modifying enzymes.

Comparison of the QRDRs of the three isolates with the corresponding region of *A. baumannii* ATCC 19,606 revealed that all of them possessed identical amino acid substitutions (pS83L in *gyrA* and pS80I in *parC*), with the previously reported role in quinolone resistance [52].

### 3.5. MLST Analysis

The two *bla*_OXA-23_-carrying XDR-AB isolates from Sofia (Aba52 and Aba176) belonged to the globally disseminated ST2, clonal complex (CC) 2 or IC2. The MLST analysis assigned the Aba190 isolate from Varna (coexistence of *bla*_OXA-23_ and *bla*_OXA-72_) to ST636, a triple-locus variant of ST2 (Table 3).

### 3.6. Phylogenomic Analysis

**Figure 2:**
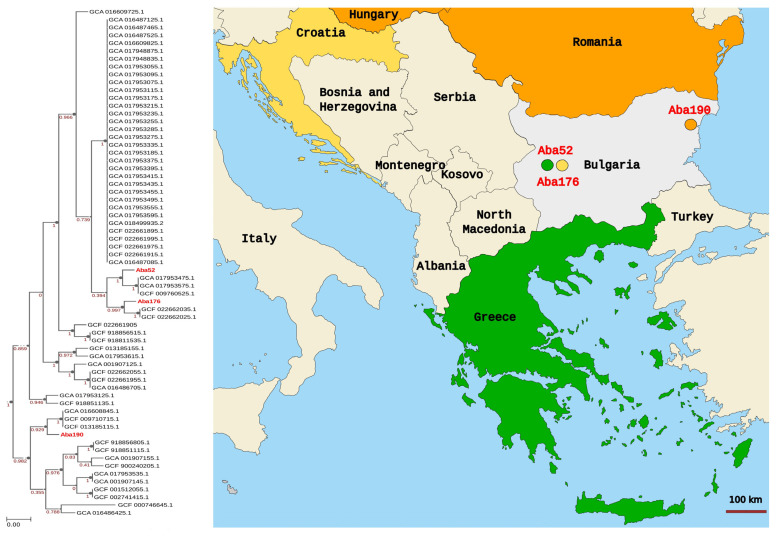
Phylogenetic tree constructed on 49 universal core gene multiple-sequence alignment of 66 colistin-susceptible *A. baumannii* clinical isolates from neighboring South-Eastern European countries. The countries of origin for the closest matches of the three isolates here were color-coded.

To analyze the local epidemiology of our isolates, a phylogenetic tree based on SNPs in 49 universal genes was built, as described above. The 66 genomes, included in the analysis were previously described [53]. They all originated from colistin-susceptible isolates. The resulting tree is presented in Figure 2.

The Aba52 isolate (Sofia, 2018) was closely related to a group of three clinical isolates from Greece, obtained in the period of 2013–2016. Aba176 (Sofia, 2019) was positioned next to two Croatian isolates recovered in 2020. Aba190 (Varna, 2019) had neighbors from Hungary (one isolate, 2017) and Romania (two isolates obtained during the period of 2017–2018).

## 4. Discussion

The investigated clinical *A. baumannii* isolates were problematic ICU pathogens. Antimicrobial susceptibility testing revealed retained activity of only two of the antibiotics studied: COL (0% resistance) and SAM (13.7% resistance). These antibiotics have been involved in the recommended therapeutic regimens of severe infections caused by MDR-AB at the respective hospitals [54]. In line with global trends, to treat severe CRAB infections in critically ill patients, such as hospital-acquired pneumonia, VAP, BSIs with severe sepsis/septic shock, combined therapy of two agents active in vitro was increasingly being used, including COL, high-dose SAM, an aminoglycoside, high-dose TGC, and high-dose minocycline [15]. Treatment recommendations for PDR-AB infections included cefiderocol if available, or triple-drug combination of COL, high-dose MEM and high-dose SAM [15,55,56]. A recent study demonstrated the clinical efficacy of rescue therapy with cefiderocol for severe BSIs and VAP caused by CRAB among Italian critically ill patients [57]. Additionally, over the past five years, there has been an increasing number of studies reporting positive results in the treatment of CRAB/MDR-AB infections with the durlobactam–sulbactam combination [58]. Durlobactam is a new member of the diazabicyclooctane class of β-lactamase inhibitors, with broad-spectrum activity against Ambler class A, C, and D β-lactamases. Sulbactam is a first generation β-lactamase inhibitor, which also possesses antibacterial activity against *A. baumannii*, due to its selective binding to the penicillin-binding proteins 1, 2, and 3 [59]. To date, the frequency of *A. baumannii* spontaneous resistance to this dual β-lactamase inhibitor combination has been low [60]. It is worth mentioning that the global prevalence of MDR-AB and XDR-AB isolates has renewed interest in the therapy with non-antibiotic options, such as lytic bacteriophages [61,62] and bacteriophage-encoded endolysin [63].

It is estimated that resistance to carbapenems alone is sufficient for *A. baumannii* to be considered a highly resistant and therapeutically problematic pathogen [64]. In the current study, the prevalence of XDR-AB isolates (78.1%) was significantly higher (*p* < 0.001) than that observed among nosocomial CRAB isolated at four Bulgarian university hospitals during the period of 2014–2016 (12.4%), as well as than their proportion (21.7%) in CRAB isolates obtained from critically ill patients undergoing renal replacement therapy at a large university hospital in Sofia, Bulgaria (2016–2018) [65,66]. Analysis of the antimicrobial resistance of the investigated CRAB isolates compared to that among the isolates from the aforementioned studies showed a chronological increase in resistance to both TOB (86.3% resistant isolates vs. 55.8% (2014–2016) and 60.9% (2016–2018); *p* < 0.001 and *p* < 0.05, respectively) and TGC (86.3% vs. 58.4% non-susceptible isolates (2014–2016), *p* < 0.001, and 69.6% resistant isolates (2016–2018)). Moreover, it is worth noting a significant decrease in SAM resistance reported in the present study (13.7%) compared to the two earlier Bulgarian studies (41.6–43.5%; *p* < 0.01–0.001) [65,66], which confirms the role of this antimicrobial agent in treatment regimens for ICU patients [15]. Our finding contrasted with the large proportion of contemporary CRAB isolates demonstrating high-level resistance to sulbactam, mainly associated with the spread of OXA-23 producers in high-prevalence geographic areas, such as China [67].

The antimicrobial resistance rates to carbapenems, aminoglycosides and fluoroquinolones in our nosocomial CRAB isolates were similar to those reported by the EURECA study including invasive CRAB isolates from 10 European countries (Albania, Croatia, Greece, Italy, Kosovo, Montenegro, Romania, Serbia, Spain and Turkey) in the period between May 2016 and November 2018, as follows: IMP, 99.6% resistance; MEM, 99.6%; AMK, 95.6%; GEN, 92.9%; TOB, 80.9%; and ciprofloxacin, 100%. The authors found 9.3% colistin-resistant isolates that were not identified in the present study [26]. Recent studies identified nosocomial colistin-resistant CRAB isolates in several neighboring Balkan countries, such as Croatia (during the period of 2013–2018), Greece (2015–2017) and Serbia (2018–2021) [53,68,69]. 

The European Antimicrobial Resistance Surveillance Network (EARS-Net) data for 2020, published by the European Center for Disease Prevention and Control (ECDC), reported a very high prevalence of invasive carbapenem-resistant *Acinetobacter* spp. (over 80%) in all Balkan countries [70]. The frequencies of distribution in ascending order were as follows: Bulgaria (82.9%), Kosovo (84.7%), Turkey (93.1%), Romania (93.3%), Greece (94.6%), Croatia (96.4%), North Macedonia (97.4%), Bosnia and Herzegovina (97.9%), Serbia (98.6%), and Montenegro (100%). This occurrence was approximately 2.5 times higher than the average value for the European Union (EU)/European Economic Area (EEA) region (38%), highlighting the Balkan region as a hotspot on the surveillance map. On the other hand, the prevalence of MDR (combined resistance to carbapenems, fluoroquinolones and aminoglycosides) *Acinetobacter* spp. exceeded 70%, with the highest values reported in Serbia (95.9%) and Croatia (95.1%), and the lowest in Kosovo (71.2%) [70]. The prevalence of invasive *Acinetobacter* spp. isolates, exhibiting combined resistance to carbapenems, fluoroquinolones and aminoglycosides, in Bulgaria was significantly higher than the average combined resistance for the EU/EEA region (72.9% vs. 34.1%; *p* < 0.001).

In the present study, the frequency of genes encoding intrinsic OXAs (OXA-51-like) was absolute. Previous studies have shown that genes for naturally occurring OXA-51-like enzymes of *A. baumannii* are localized on the chromosome and their expression is regulated by IS*Aba1* [23], found in almost all our isolates. Insertion of IS*Aba1* at the start of the promotor region, immediately upstream the structural gene, results in carbapenemase activity of these enzymes [24,71]. In addition, all CRAB isolates harbored genes for acquired CHDLs as follows: 71 were identified to be positive for *bla*_OXA-23-like_; one isolate was *bla*_OXA_-_24/40-like_-positive (obtained from an ICU patient treated in the SM-V) and one isolate carried both *bla*_OXA-23-like_ and *bla*_OXA-24/40-like_ genes (SM-V). Afterwards, the genome analysis revealed the presence of *bla*_OXA-23_ (OXA-23-like family CHDLs), *bla*_OXA-66_ (OXA-51-like family CHDLs) and *bla*_OXA-72_ (OXA-24/40-like family CHDLs).

For the first time in Bulgaria, Stoeva et al. reported the clonal spread of 29 carbapenem-resistant OXA-23-producing *A. baumannii* isolates obtained from inpatients at a university hospital in Pleven during the period of 1999–2006 [72]. The *bla*_OXA-23_ gene, associated with the upstream-located IS*Aba1*, was identified as the mechanism responsible for carbapenem resistance in all isolates. Thereafter, widespread dissemination of clonally-related OXA-23-producing CRAB isolates was also found at large hospitals in other cities of the country [65,73,74,75,76]. *bla*_OXA-23_ is the predominant carbapenemase-coding gene, being present in two-thirds of the CRAB isolates from countries of all continents [26,71]. Further, this gene has been reported to be the most prevalent antimicrobial resistance determinants among CRAB isolates in several studies in the Balkan region, including Croatia [68], Greece [69], Romania [77], and Turkey [78], as well as in the EURECA study (67.7%) conducted on invasive CRAB isolates from 10 European countries, most of them, again, from the Balkans [26]. The *bla*_OXA-23_ gene is either located on the chromosome or on plasmids and is associated with different genetic structures [22,79].

In the present study, the frequency of distribution of *bla*_OXA-24/40-like_ genes found only in SM-V isolates was significantly lower than that detected in earlies CRAB isolates (2014–2016) from four Bulgarian large hospitals (2.7% vs. 66.4%; *p* < 0.001) [65]. In accordance with our finding, in the cited study, OXA-24/40-like CHDLs were reported as the most common mechanism, conferring carbapenem resistance among CRAB isolates in SM-V. The isolates were clonally related and approximately 86.4% of them were identified as *bla*_OXA-24/40-like_-positive [65]. The first Bulgarian *bla*_OXA-24-like_-carrynig CRAB isolates were identified in 2012 [80]. Through sequencing techniques, OXA-72 carbapenemase, a variant of OXA-24/40-like CHDLs, was later found in the country [65,76]. One of our two *bla*_OXA-24/40-like_-postive isolates (Aba190) was also determined as an OXA-72 producer by WGS. The OXA-72 carbapenemase was reported in the Balkan region for the first time in Serbia in 2016 [81]. Moreover, 30% of the CRAB isolates in the EURECA study, predominantly from Serbia, harbored *bla*_OXA-72_ [26]_._ Additionally, the coexistence of *bla*_OXA-72_ and *bla*_NDM-1_ was found in four ST492 isolates from a Serbian hospital. Like OXA-23, OXA-72 CHDLs (chromosomally or plasmid-encoded) have been reported worldwide, including in Taiwan, China, Brazil, Colombia, the USA, France, Poland, Italy, Belgium, Lithuania, Sweden and Croatia [71,79].

The WGS of three selected *A. baumannii* isolates revealed that the resistance to carbapenems was mainly mediated by the presence of CHDLs (OXA-23, OXA-66 and OXA-72). Moreover, Aba52 and Aba176 also harbored IS*Aba24* and IS*Aba125* insertion sequences that were positioned in the promotor regions of the *bla*_ADC-25_ and *bla*_OXA-23_ genes, likely contributing to their transcriptional upregulation. Previous studies have highlighted IS*Aba125* as a particularly efficient element in this regard due to its better promoter sequence compared to IS*Aba1* [82]. The Aba190 isolate compensates for the absence of these elements with the presence of a *bla*_OXA-72_ gene, located on a small plasmid. This finding raised concerns since OXA-72 can be easily transmitted via horizontal gene transfer due to its localization. Moreover, the plasmid also harbored an IS*Aba31* element, which creates an additional possibility for gene transfer via recombinational events between insertion sequences.

Aminoglycosides have traditionally been included in therapeutic regimens for the treatment of infections caused by CRAB [15]. Unfortunately, aminoglycoside resistance has also increased in the recent years [70]. A total of 86.3% of the isolates tested in this study demonstrated high-level resistance to the three aminoglycosides (AMK, GEN and TOB), with MIC values above 256 mg/L. Methylation of the 16S rRNA in the 30S ribosomal subunit is recognized as the most common molecular mechanism, conferring high-level resistance to most clinically useful aminoglycosides [3,43]. All high-level-resistant *A. baumannii* isolates in this study were identified to be ArmA 16S rRNA methylase producers. A previous study found 32.9% ArmA-producing CRAB isolates among MDR-AB isolates obtained from ICU patients at a Bulgarian university hospital during the period of 2010–2011 [74]. The authors reported plasmid localization of the encoding gene, as well as coexistence of *armA* and *bla*_OXA-23_. Moreover, the co-production of ArmA 16S rRNA methylase and OXA-23 carbapenemase was determined as the most prevalent mechanism conferring MDR in clinical *A. baumannii* isolates in many countries worldwide, such as Italy, Greece, China, South Korea, India, Yemen etc. [69,71,83].

Similar to other pathogenic bacteria, class 1, 2 and 3 integrons that harbored *sul* genes, responsible for sulfonamide resistance, have been found in nosocomial MDR-AB isolates [84,85]. As described above, all SXT-resistant CRAB isolates in the present study were *sul1*-positive and the three WGS-subjected ones were confirmed as class-1-carrying isolates.

Two of the three WGS analyzed XDR-AB isolates belonged to the high-risk ST2 (CC2, IC2) and one of them was classified into ST636 (a triple-locus variant of ST2). ST2 is a CRAB lineage that has been reported from various parts of the world [24,26,79,86]. Of note, ST2 was found to be the most common ST among the CRAB isolates (2016–2018), included in the EURECA multicenter study (67.7%) [26]. Additionally, a large-scale WGS-based study determined ST2 as the most prevalent ST in CRAB isolates (2013–2017) from three Mediterranean countries as follows: Greece (93.5%), Italy (82.9%) and Israel (40%) [86]. Recently, *Cherubini* et al. investigated *A. baumannii* isolates recovered from the blood cultures of 43 critically ill COVID-19 patients treated in the ICU ward of a hospital in Pescara, Central Italy. Interestingly, all isolates demonstrated a remarkable antimicrobial resistance, including MEM and COL, and belonged to ST2 high-risk international clone [87]. ST2 was predominant (58.1%), followed by ST636 (14%), in clinical *A. baumannii* isolates collected across Belgium 2014–2017. The collection included CRAB, MDR-AB, XDR-AB, and even PDR-AB isolates [79]. Recently, Gajic et al. reported an outbreak of BSI in preterm neonates caused by *A. baumannii bla*_OXA-66_/*bla*_OXA-72_/ST636 in a neonatal ICU in Serbia [88]. Moreover, OXA-72-producing CRAB isolates, belonging to ST636, were previously found in a nationwide study conducted in Serbia [89].

The phylogenomic analysis revealed that the Bulgarian isolates were closely related to those collected from Greece, Croatia and Romania. These results suggest that human migration and travel facilitate the spread of high-risk CRAB clones in the Balkans.

## 5. Conclusions

The studied nosocomial CRAB isolates also demonstrated cross-resistance to antimicrobial agents from other classes, which was the basis for categorizing them as MDR-AB (21.9%) or XDR-AB (78.1%). Antimicrobial treatment choices for these problematic isolates were severely limited. There were only a few effective options available, such as COL and SAM and, rarely, SXT.

Production of acquired CHDLs, predominantly OXA-23, was found in all isolates. The WGS revealed the presence of different ISs, such as IS*Aba24*, IS*Aba31*, IS*Aba125*, IS*Vsa3*, IS*17*, and IS*6100*, which, in addition to the most common IS*Aba1*, allow *A. baumannii* the high ability to capture and mobilize ARGs. Moreover, the emergence of OXA-72, associated with a plasmid and transposable mobile genetic elements, such as IS*Aba31*, increases the risk of horizontal transfer of ARGs among nosocomial *A. baumannii* isolates, as well as other Gram-negative pathogens in Bulgaria. A geographical difference between the isolates from Sofia and Varna was established, both in terms of the identified antimicrobial resistance determinants and mobile genetic elements, as well as their ST affiliations (high-risk ST2 and ST636 belonging to IC2).

To the best of our knowledge, this is the first WGS-based resistome analysis of *A. baumannii* conducted in Bulgaria. The implementation of a rapid technique, such as next-generation sequencing, to identify resistomes and clonal relatedness of problematic pathogens in hospitals could be useful for effective antimicrobial stewardship programs. Our results show a widespread dissemination of XDR-AB isolates, carrying a variety of ARGs in Bulgarian ICU settings, which highlights the crucial need for nationwide surveillance, especially in the conditions of extensive antibiotic usage during the COVID-19 pandemic.

## Figures and Tables

**Table 1:** Oligonucleotides used as primers for PCR amplification of antimicrobial resistance genes in *A. baumannii*.

Primer Pairs	Target	Sequence (5′–3′) ^a^	Product Size (bp)	Ta (°C)	Source
OXA23LF ^b^ OXA23LR ^b^	OXA-23-like CHDLs	GATCGGATTGGAGAACCAGA ATTTCTGACCGCATTTCCAT	501	58.3	[37]
OXA24LF ^b^ OXA24LR ^b^	OXA-40/24-like CHDLs	GGTTAGTTGGCCCCCTTAAA AGTTGAGCGAAAAGGGGATT	246	58.3	[37]
OXA51LF ^b^ OXA51LR ^b^	OXA-51-like CHDLs	TAATGCTTTGATCGGCCTTG TGGATTGCACTTCATCTTGG	353	58.3	[37]
OXA58LF ^b^ OXA58LR ^b^	OXA-58-like CHDLs	AAGTATTGGGGCTTGTGCTG CCCCTCTGCGCTCTACATAC	599	58.3	[37]
OXA143F OXA143R	OXA-143-like CHDLs	TGGCACTTTCAGCAGTTCCT TAATCTTGAGGGGGCCAACC	146	60	[38]
OXA235F OXA235R	OXA-235-like CHDLs	TTGTTGCCTTTACTTAGTTGC CAAAATTTTAAGACGGATCG	768	56	[39]
IS*Aba1*F IS*Aba1*R	IS*Aba1*	CACGAATGCAGAAGTTG CGACGAATACTATGACAC	549	50	[40]
IMP-F ^c^ IMP-R ^c^	IMP-type MBLs	GGAATAGAGTGGCTTAAYTCTC GGTTTAAYAAAACAACCACC	232	58.5	[41]
VIM-F ^c^ VIM-R ^c^	VIM-type MBLs	GATGGTGTTTGGTCGCATA CGAATGCGCAGCACCAG	390	58.5	[41]
NDM-F ^c^ NDM-R ^c^	NDM-type MBLs	GGTTTGGCGATCTGGTTTTC CGGAATGGCTCATCACGATC	621	58.5	[41]
KPC-F KPC-R	KPC-type class A carbapenemases	CTGTCTTGTCTCTCATGGCC CCTCGCTGTRCTTGTCATCC	796	60	[42]
ArmA-F ArmA-R	ArmA 16S rRNA methylase	ATTCTGCCTATCCTAATTGG ACCTATACTTTATCGTCGTC	315	56	[43]
Sul1-F Sul1-R	Dihydropteroate synthase type I	ACGGTGTTCGGCATTCT TTTGAAGGTTCGACAGC	581	53	[44]

PCR, polymerase chain reaction; Ta, annealing temperature; F, forward primer; R, reverse primer; CHDL, carbapenem-hydrolyzing class D β-lactamase; IS, insertion sequence; MBL, metallo-β-lactamase. ^a^ Y = C or T, R = G or A; ^b^ Primer pairs used in a multiplex PCR for CHDL-encoding genes; ^c^ Primer pairs used in a multiplex PCR for MBL-encoding genes.

**Table 2:** Phenotypic and genotypic characteristics of the whole-genome sequencing-subjected *A. baumannii* isolates.

Isolate No.	Origin	Hospital	Year	Antimicrobial Agent: MIC [mg/l] (Interpretation)									PCR-Detected ARGs
				IMP	MEM	AMK	GEN	TOB	LVX	TGC	SXT	COL	
Aba52	Blood	SIR-S	2018	>32 (R)	>32 (R)	>256 (R)	>256 (R)	>256 (R)	24 (R)	2 (R)	>256 (R)	1 (S)	*bla*_OXA-51-like_, *bla*_OXA-23-like_, *armA*, *sul1*
Aba176	TBA	SIR-S	2019	16 (R)	>32 (R)	>256 (R)	>256 (R)	>256 (R)	16 (R)	1.5 (R)	>256 (R)	1 (S)	*bla*_OXA-51-like_, *bla*_OXA-23-like_, *armA*, *sul1*
Aba190	TBA	SM-V	2019	>32 (R)	>32 (R)	48 (R)	8 (R)	6 (R)	8 (R)	1.5 (R)	128 (R)	1 (S)	*bla*_OXA-51-like_, *bla*_OXA-23-like_, *bla*_OXA-24/40-like_, *sul1*

TBA, tracheobronchial aspirate; SIR-S, University Hospital ‘St. Ivan Rilski, Sofia; SM-V, University Hospital ‘St. Marina’, Varna; MIC, minimum inhibitory concentration; IMP, imipenem; MEM, meropenem; AMK, amikacin; GEN, gentamicin; TOB, tobramycin; LVX, levofloxacin; TGC, tigecycline; SXT, trimethoprim–sulfamethoxazole; COL, colistin; S, susceptible; R, resistant; PCR, polymerase chain reaction; ARGs, antimicrobial resistance genes.

**Table 3:** Whole genome-based characterization of the studied *A. baumannii* isolates.

Isolate	Genome Size (Mbp)	GC%	N50 (bp)	No. of Contigs	ST	Alleles						
						*cnp60*	*fusA*	*gltA*	*pyrG*	*recA*	*rplB*	*rpoB*
Aba52	3.96	38.9	122,028	109	2	2	2	2	2	2	2	2
Aba176	3.97	38.9	83,512	131	2	2	2	2	2	2	2	2
Aba190	4.14	38.9	92,248	182	636	2	1	2	2	2	1	1

ST, sequence type determined according to the Pasteur Multilocus Sequence-Typing scheme.

**Table 4:** Antimicrobial resistance genes (ARGs) and mobile genetic elements (MGEs) found in the *A. baumannii* isolates studied.

ARGs/MGEs	Aba52	Aba176	Aba190
MGEs	IS*Aba24*, IS*Aba125*, IS*Vsa3*, IS*6100*	IS*Aba24*, IS*Aba125*, IS*Vsa3*, IS*6100*	Tn*6018*, IS*Aba31*, IS*17*
β-Lactam Resistance	*bla*_ADC-25_, *bla*_OXA-23_, *bla*_OXA-66_	*bla*_ADC-25_, *bla*_OXA-23_, *bla*_OXA-66_	*bla*_ADC-25_, *bla*_OXA-23_, *bla*_OXA-66_, *bla*_OXA-72_
Aminoglycoside Resistance	*armA*, *aph(3″)-Ib*, *aph(6)-Id*, *aac(3)-Ia*, *ant(3″)-IIa*, *aadA1*	*armA*, *aph(3′)-VIa*, *aph(3″)-Ib*, *aph(6)-Id*, *aac(3)-Ia*, *ant(3″)-IIa*, *aadA1*	*aph(3′)-Ia*, *aph(3′)-VIa*, *aac(3)-Ia*, *ant(3″)-IIa*, *aadA1*
Macrolide Resistance	*mph(E)*, *msr(E)*	*mph(E)*, *msr(E)*	*mph(E)*, *msr(E)*
Others	*sul1*, *tet(B)*, *catA1*	*sul1*, *tet(B)*, *catA1*	*sul1*

IS, insertion sequence; Tn, transposon.

## Data Availability

Whole-genome shotgun sequencing projects of the *A. baumannii* Aba52, Aba176 and Aba190 isolates have been deposited in GenBank under Accession numbers JARNMV000000000, JARNMW000000000 and JAR NMX000000000, respectively.

## Supplementary Materials

The following supporting information can be downloaded at: https://www.mdpi.com/article/10.3390/microorganisms11040875/s1, Table S1: Genomes of colistin-susceptible *A. baumannii* isolates (*n* = 66) used to build a phylogenetic tree; Table S2: Antimicrobial susceptibility of the studied carbapenem-resistant *A. baumannii* isolates (*n* = 73).
