# Supplementary material for: Phenotypic and Molecular Characteristics of Carbapenem-Resistant Acinetobacter baumannii Isolates from Bulgarian Intensive Care Unit Patients
Source: Microorganisms. 2023 Mar 29;11(4):875. doi: 10.3390/microorganisms11040875 (PMC10141887; doi:10.3390/microorganisms11040875)
Supplement: Supplementary file 1 [file microorganisms-11-00875-s001.zip › microorganisms-2279131-supplementary.pdf]

***Article:*** Phenotypic and Molecular Characteristics of Carbapenem-Resistant *Acinetobacter baumannii* Isolates from Bulgarian Intensive Care Unit Patients (Tanya V. Strateva *et al.*)

**Table S1.** Genomes of colistin-susceptible *A. baumannii* isolates ($n$ = 66) used to build a phylogenetic tree.

| NCBI Accession Number | Location | Isolation Year |
|---|---|---|
| GCA_001512055.1 | Greece | 2002 |
| GCA_001907125.1 | Croatia | 2009 |
| GCA_001907145.1 | Croatia | 2003 |
| GCA_001907155.1 | Croatia | 2013 |
| GCA_002741415.1 | Greece | 2002 |
| GCA_009710715.1 | Romania | 2018 |
| GCA_009760525.1 | Greece | 2016 |
| GCA_013185115.1 | Romania | 2017 |
| GCA_013185135.1 | Romania | 2017 |
| GCA_013185155.1 | Romania | 2017 |
| GCA_016486425.1 | Greece | 2016 |
| GCA_016486705.1 | Hungary | 2012 |
| GCA_016487085.1 | Croatia | 2014 |
| GCA_016487125.1 | Croatia | 2014 |
| GCA_016487465.1 | Greece | 2014 |
| GCA_016487525.1 | Greece | 2014 |
| GCA_016608845.1 | Hungary | 2017 |
| GCA_016609725.1 | Romania | 2011 |
| GCA_016609825.1 | Greece | 2014 |
| GCA_017948835.1 | Greece | 2015 |
| GCA_017948875.1 | Greece | 2015 |
| GCA_017953055.1 | Greece | 2016 |
| GCA_017953075.1 | Greece | 2016 |
| GCA_017953095.1 | Greece | 2016 |
| GCA_017953115.1 | Greece | 2016 |
| GCA_017953125.1 | Greece | 2015 |
| GCA_017953155.1 | Greece | 2015 |
| GCA_017953175.1 | Greece | 2015 |
| GCA_017953185.1 | Greece | 2014 |
| GCA_017953215.1 | Greece | 2014 |
| GCA_017953235.1 | Greece | 2016 |
| GCA_017953255.1 | Greece | 2016 |
| GCA_017953275.1 | Greece | 2016 |
| GCA_017953285.1 | Greece | 2015 |
| GCA_017953315.1 | Greece | 2016 |
| GCA_017953335.1 | Greece | 2015 |

| | | |
|---|---|---|
| GCA_017953355.1 | Greece | 2015 |
| GCA_017953375.1 | Greece | 2015 |
| GCA_017953395.1 | Greece | 2015 |
| GCA_017953415.1 | Greece | 2015 |
| GCA_017953435.1 | Greece | 2015 |
| GCA_017953455.1 | Greece | 2015 |
| GCA_017953475.1 | Greece | 2014 |
| GCA_017953495.1 | Greece | 2014 |
| GCA_017953515.1 | Greece | 2014 |
| GCA_017953535.1 | Greece | 2014 |
| GCA_017953555.1 | Greece | 2014 |
| GCA_017953575.1 | Greece | 2013 |
| GCA_017953595.1 | Greece | 2014 |
| GCA_017953615.1 | Greece | 2013 |
| GCA_018499935.2 | Hungary | 2018 |
| GCA_022661895.1 | Croatia | 2020 |
| GCA_022661905.1 | Croatia | 2020 |
| GCA_022661915.1 | Croatia | 2020 |
| GCA_022661955.1 | Croatia | 2020 |
| GCA_022661975.1 | Croatia | 2020 |
| GCA_022661995.1 | Croatia | 2020 |
| GCA_022662025.1 | Croatia | 2020 |
| GCA_022662035.1 | Croatia | 2020 |
| GCA_022662055.1 | Croatia | 2020 |
| GCA_900240205.1 | Bosnia and Herzegovina | 2011 |
| GCA_918811535.1 | Romania | 2018 |
| GCA_918851115.1 | Romania | 2018 |
| GCA_918851135.1 | Romania | 2018 |
| GCA_918856515.1 | Romania | 2018 |
| GCA_918856805.1 | Romania | 2018 |

NCBI, National Center for Biotechnology Information. *Note:* Isolates closely related to Aba52, Aba176 and Aba190 are color-coded.

**Table S2.** Antimicrobial susceptibility of the studied carbapenem-resistant *A. baumannii* isolates (*n* = 73).

| Antimicrobial agents | MIC range (mg/L) | $MIC_{50}$ (mg/L) | $MIC_{90}$ (mg/L) | Number (percent) of isolates [a] | | |
|---|---|---|---|---|---|---|
| | | | | S | I | R |
| Imipenem | 16 – >32 | >32 | >32 | 0 (0) | 0 (0) | 73 (100) |
| Meropenem | 16 – >32 | >32 | >32 | 0 (0) | 0 (0) | 73 (100) |
| Amikacin | 2 – >256 | >256 | >256 | 1 (1.4) | 0 (0) | 72 (98.6) |
| Gentamicin | 0.75 – >256 | >256 | >256 | 8 (11) | 0 (0) | 65 (89) |
| Tobramycin | 0.5 – >256 | >256 | >256 | 10 (13.7) | 0 (0) | 63 (86.3) |
| Levofloxacin | 4 – >32 | 12 | >32 | 0 (0) | 0 (0) | 73 (100) |
| SXT (1:19) | 0.25 – >256 | 32 | >256 | 17 (23.3) | 2 (2.7) | 54 (74) |
| Tigecycline [b] | 0.5 – >256 | 1.5 | 32 | 10 (13.7) | 0 (0) | 63 (86.3) |
| Colistin | 0.5 – 1 | 1 | 1 | 73 (100) | 0 (0) | 0 (0) |
| Ampicillin-sulbactam (1:1) | N/A | N/A | N/A | 51 (69.9) | 12 (16.4) | 10 (13.7) |

MIC, minimum inhibitory concentration; $MIC_{50/90}$, MICs at which 50% and 90% of the isolates are inhibited, respectively; S, susceptible; I, intermediate; R, resistant; SXT, trimethoprim-sulfamethoxazole; N/A, not applicable.

[a] According to European Committee on Antimicrobial Susceptibility Testing (EUCAST) 2019 criteria [34], except for ampicillin-sulbactam that was interpreted according to Clinical and Laboratory Standards Institute (CLSI) 2019 criteria [35].

[b] Interpreted according to the criteria for *Enterobacterales*.
